# Q&A with Editorial Board Member Professor Nathalie Katsonis

**DOI:** 10.1038/s42004-022-00730-3

**Published:** 2022-09-22

## Abstract

Hear from Professor Nathalie Katsonis on her academic journey and passions, her thoughts on the future directions of chemical research, and her experience of being an Editorial Board Member for *Communications Chemistry*.

Nathalie Katsonis received her MSc (2001) and PhD (2004) degrees from the University Pierre et Marie Curie (Paris, France). Her investigations of the interplay between motion, light and molecular machines started in the group of Ludovic Jullien, where she synthesized an analogue of the chromophore that initiates the flagellar movement of purple bacteria, and studied its photochemistry. For her postdoctoral research she moved to the group of Ben Feringa to investigate chirality and order in supramolecular assemblies.

**Figure:**
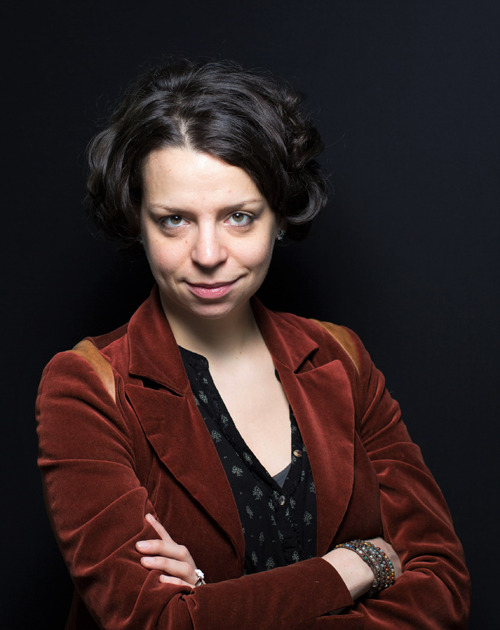
Nathalie Katsonis

Her independent career started in 2007 as Associate Researcher for the French National Center for Scientific Research. In 2008 she was invited back to Groningen to work with Feringa on artificial molecular motors and switches. In 2011 she took up a tenure-track position at the MESA + Institute for Nanotechnology at the University of Twente (the Netherlands), where she was promoted to Associate Professor in 2014 and to Full Professor in ‘Bio-inspired and Smart Materials’ in 2017. In March 2020 she joined the Stratingh Institute of Chemistry of the University of Groningen (the Netherlands), as Professor of chemistry, founding the group ‘Active Molecular Systems and Materials’.

Katsonis has led the way in transmitting directed molecular motion across length scales, with a special focus on the role of chirality and on the effects of mechanically relevant motion of molecular machines. She has recently developed an interest in the motion of supramolecular compartments in fluids. A central objective of this program is to create droplets, vesicles, and other microcompartments that can move autonomously by using metabolic energy. Her achievements have provided the underpinnings for increasingly complex functionalities in dynamic and ultimately life-like supramolecular materials.

Why did you choose to be a scientist?

Becoming a scientist has always been evident for me, so I don’t believe it was a conscious decision on my part. I did, however, choose chemistry deliberately. My mother is both a mathematician and economist, and my father is a physicist, so I believe that by choosing chemistry I demonstrated originality! Chemistry was my strongest subject, but I also enjoyed it more because I felt that if I created the objects of my own study, I would have more room for creativity. This impression was ultimately confirmed.

In the French system at the time, engineering schools, known as “Grandes Ecoles”, were the typical entry point for students interested in science. I succeeded the entrance examinations of Chimie Paris, which is luckily for me a very research-oriented school. The focus suited me well, and I made some excellent friends there. I felt truly fulfilled after enrolling in the Master of condensed matter at the University that was at the time called “Université Pierre et Marie Curie”. Among other teachers, I had a special appreciation for Ludovic Jullien; his views have irreversibly shaped the way I see chemistry and I published my first paper with him too.

What scientific development are you currently most excited about?

There are so many exciting things happening now — I feel it’s a golden age for chemistry. Developing RNA vaccines and all the associated research and development would be my most obvious response. There is a great deal of chemistry involved, as the RNA is fragile and must be enclosed in a synthetic lipid-based fat bubble. The need for lipid transfer bubbles has prompted a wealth of exciting research on the synthesis and self-assembly features of lipids.

Active and passive mass transport properties—at the molecular level and above—are also the focus of new research sparked by this development. Mass transport has, of course, been extensively studied at the macroscopic level and in bulk systems. In contrast, using molecular design allows controlling mass transport in supramolecular systems and guiding interactions between large ensembles of molecules with specific functions—what we refer to as complex molecular systems. My personal feeling is that controlling active mass transport at intermediate length scales offers tremendous opportunities for the future.

What direction do you think your research field should go in?

I believe that the development of artificial intelligence will have a significant impact on all research fields, including chemistry. We are currently experiencing a paradigm shift, where we go from a traditional way of doing chemistry, towards automatized laboratories where the production of large data sets will be coupled to artificial intelligence, to aid discovery. The effectiveness of these approaches will also facilitate major steps towards sustainable formulations and a circular chemical industry. Such a change of paradigm impacts academic research, chemical industries, and also education – it implies that we will change the minds of educators, and altogether it’s a very exciting time for the chemistry community. We can’t be sure where it will lead us, but there is a general trend and every day we discover new things along the way.

What attracted you to becoming an Editorial Board Member for *Communications Chemistry*? What have you gotten out of the experience?

I view my position on the Editorial Board as a chance to contribute to shaping the future of chemistry, if only on a small scale. I specifically chose to contribute to *Communications Chemistry* after being asked by a former PhD student of Steve Fletcher, who was Associate Editor at the time. I always have had relations of trust with Andrew and therefore I was inclined to accept the invitation. I see my task as contributing to building a community in the fields of molecular machines and systems chemistry and bringing people together, so they can become more than the sum of their parts.

How do your editorial responsibilities integrate with your academic role?

As scientists we bear responsibility towards society—we educate, we communicate, and also we work on scientific problems that are relevant for both chemistry and society. If one is isolated, it is not always easy to have a feeling for what is relevant. Editorial responsibilities keep you alert in terms of broader interests, what is relevant, where chemistry should go. Beyond my own community, the work keeps me engaged with the whole chemistry community.

What do you see as the role of *Communications Chemistry* in the scientific community?

Within the Nature Portfolio, *Communications Chemistry* embraces the possibility to do things differently—it promotes open access publication, maintains close collaboration between Editorial Board Members and professional editors to exchange good practices and different ways of doing things, offers the possibility to publish valuable, solid and sound chemistry. The journal also tries original initiatives, like supporting young investigators financially to attend conferences or acknowledging reviewer’s work with reviewer of the month nominations.

*This interview was conducted by the editors of Communications Chemistry*.

